# Sex‐related differences in the peripheral vascular response to hypoxia: Implications in health and relevance for obstructive sleep apnoea

**DOI:** 10.1113/EP092842

**Published:** 2026-04-29

**Authors:** Dain W. Jacob, Sara E. Mascone, Natasha G. Boyes, Anna M. Gonsalves, Jacqueline K. Limberg

**Affiliations:** ^1^ Department of Nutrition & Exercise Physiology University of Missouri Columbia Missouri USA; ^2^ Department of Kinesiology, School of Public Health University of Maryland College Park Maryland USA; ^3^ Baltimore Geriatric Research, Education, and Clinical Center, Baltimore Veterans Affairs Medical Center Baltimore Maryland USA

**Keywords:** adrenergic, blood pressure, hypoxia, intermittent hypoxia, women

## Abstract

The incidence and presentation of obstructive sleep apnoea, as well as hypertension and cardiovascular disease, have been reported to differ by sex. It is thus reasonable to propose mechanisms contributing to subsequent pathology differ by sex. This concept is supported from a pre‐pathological perspective, as sex differences have been observed in the vascular response to hypoxia both alone and during concomitant hypoxia and sympathetic activation. However, the mechanisms governing the vascular response to hypoxia in females compared to males have only recently been examined, with the preponderance of data in predominantly male or all‐male cohorts. Determining the sex‐specific mechanisms contributing to the foundational vascular response to hypoxia is essential to the development of targeted therapeutics. Herein we outline literature in support of vascular sex‐related differences in the acute response to both intermittent and steady‐state hypoxia, with an emphasis on the healthy state. However, questions remain to bridge the gap between preclinical observations to pathological conditions, such as the role of sex hormones and adrenergic receptors, with increasing adiposity, age and chronic disease (e.g., sleep apnoea).

## Abstract (highlights)

- **What is the topic of this review?**
  Herein we outline literature supporting vascular sex‐related differences in the acute response to intermittent and steady‐state hypoxia, emphasizing the healthy state.
- **What advances does it highlight?**
  Mechanisms governing sex‐related differences in the vascular response to hypoxia have only recently been examined, with the preponderance of data in predominantly male cohorts. Determining sex‐specific mechanisms contributing to the foundational vascular response to hypoxia is essential to the development of targeted therapeutics. Emphasizing work from our group(s), we seek to spark new research ideas with the goal of alleviating disproportionately poor outcomes in females in conditions of hypoxia (e.g., obstructive sleep apnoea).

## 1 INTRODUCTION

Hypoxia is both a physiological and a pathological condition characterized by a mismatch in arterial oxygen supply and metabolic demand of tissue. In medical conditions where hypoxia is observed, such as obstructive sleep apnoea (OSA), cardiovascular disease (CVD) risk is prevalent (Colafella & Denton, 2018). The impact of hypoxia on CVD risk is dose‐dependent, such that the frequency, duration and severity of exposure to hypoxia are predictive of subsequent cardiovascular‐related mortality (Azarbarzin et al., 2019).

The incidence and presentation of OSA (Quintana‐Gallego et al., 2004; Roca et al., 2015), as well as hypertension and CVD (Colafella & Denton, 2018), have been reported to differ by sex. OSA is traditionally considered a ‘male disease’ (Wahner‐Roedler et al., 2007; Wimms et al., 2016). Despite lower frequency and severity of OSA in females compared to males (O'Connor et al., 2000), the cardiovascular consequences are the same, if not greater, in females (Wimms et al., 2016). Females with OSA are at increased risk of hypertension, incident heart failure and cardiovascular mortality compared to males (Drager et al., 2006; Mokhlesi et al., 2016; Quintana‐Gallego et al., 2004; Roca et al., 2015), and exhibit stronger associations between OSA severity and vascular dysfunction (Faulx et al., 2004; Randby et al., 2013). Furthermore, there are data to support that females with OSA take more medications than males and are more likely to be resistant to pharmacological interventions (Basoglu & Tasbakan, 2018; Kim et al., 2006; Mozaffarian et al., 2015). It is thus reasonable to propose that mechanisms contributing to subsequent pathology differ by sex (Bock et al., 2023).

Hypoxaemia creates a reduction in the arterial supply of oxygen to metabolically active tissues, including skeletal muscle (Joyner & Casey, 2014). The skeletal muscle vasculature must, in turn, dilate to increase blood flow and maintain net oxygen delivery (Joyner & Casey, 2014). Sex‐related differences have been observed in the vascular response to hypoxia both alone (Casey et al., 2014) and during concomitant hypoxia and sympathetic activation (i.e., cold pressor test) (Jacob et al., 2021). Despite discernible sex differences, the preponderance of data examining mechanisms governing the vascular response to hypoxia come from predominantly male or all‐male cohorts (Blauw et al., 1995; Richardson et al., 1967; Weisbrod et al., 2001; Wilkins et al., 2008). Determining the sex‐specific mechanisms contributing to the foundational vascular response to hypoxia is essential to the development of targeted therapeutics. Thus, the aim of this brief review is to highlight recent work from our group(s) examining sex‐related differences in the vascular response to both intermittent and steady‐state hypoxia.

## 2 INTERMITTENT HYPOXIA

OSA is a clinical condition characterized by exposure to hypoxia through repeated cessations or obstructions in breathing (i.e., apnoeas) resulting in a reduction in arterial oxygen. Patients with OSA are exposed to a chronic, intermittent hypoxic stimulus during sleep, with resulting nocturnal desaturations having profound physiological effects. Upon even brief, intermittent exposure to hypoxia, a reflex increase in muscle sympathetic nerve activity (MSNA) occurs which persists upon return to room air (Cutler et al., 2004a, 2004b; Jouett et al., 2017; Leuenberger et al., 2005; Morgan et al., 1995; Stuckless et al., 2020; Tamisier et al., 2011; Xie et al., 2000). In alignment, elevated daytime MSNA is observed in patients with OSA (Maier et al., 2022; Quarti‐Trevano et al., 2021; Somers et al., 1995) with sympathetic overactivity directly contributing to elevations in blood pressure in patients with OSA (Narkiewicz et al., 1998). An increase in sympathetic nervous system activity causes a proportional increase in neurotransmitter release (i.e., noradrenaline) from the sympathetic nerve terminal. Neurotransmitters bind post‐synaptic α‐adrenergic receptors to promote smooth muscle contraction, which leads to vasoconstriction, increased peripheral resistance and, subsequently, increased blood pressure. Chronic blood pressure elevation is the greatest contributor to the development of CVD (Arnett et al., 2019). Notably, elevated MSNA is a hallmark of treatment‐resistant hypertension (Grassi et al., 2025) and is predictive of mortality in CVD (Barretto et al., 2009).

Given the strong potential for sex‐specific mechanisms of OSA pathophysiology, it is perhaps surprising that the increase in MSNA following intermittent hypoxia exposure (Jacob et al., 2020), as well as the peak MSNA response to hypoxia (Jones et al., 1999; Miller et al., 2019; Sayegh et al., 2022), does not differ by sex. Notably, the vascular response to sympathetic activation following hypoxic exposure is attenuated in healthy young females. For example, just three maximal voluntary apnoeas (three repeated desaturations) are enough to elicit a reduction in forearm blood flow in healthy young males, but blood flow is preserved in females (Patel et al., 2014) – a potentially beneficial response that may be lost with ageing and/or obesity.

While there remains a paucity of mechanistic data underlying the sex‐specific vascular responses to hypoxia in humans, some preclinical work is available. A prolonged intermittent hypoxia exposure spanning 12 h per day for 7 days elicited an increase in blood pressure in male rats (Hinojosa‐Laborde & Mifflin, 2005). In this same investigation, female rats were protected from the intermittent hypoxia‐mediated increase in blood pressure (Hinojosa‐Laborde & Mifflin, 2005). Most notably, the ‘protective’ effect of female sex on the blood pressure response to intermittent hypoxia was lost with ovariectomy (i.e., elimination of ovarian hormones) (Hinojosa‐Laborde & Mifflin, 2005). In humans, premenopausal bilateral oophorectomy increases OSA risk two‐fold (Mielke et al., 2023).

Although intermittent hypoxia as an experimental model does not encompass the totality of OSA pathophysiology (Mateika, 2019), the data from Hinojosa‐Laborde and colleagues (Hinojosa‐Laborde & Mifflin, 2005), in combination with work in healthy humans (Patel et al., 2014), demonstrate dichotomous roles of sex in the peripheral vascular and blood pressure response to intermittent hypoxia and support a potential role for ovarian hormones in mediating vascular protection in females (Hinojosa‐Laborde & Mifflin, 2005). More recently, our groups (Jacob et al., 2020; Mascone et al., 2025) have expanded these data to humans, showing the blood pressure response following 30 min of acute intermittent hypoxia is increased in young males but not young females.

Utilizing lower body negative pressure to provoke increases in MSNA, Stuckless and colleagues observed the vasoconstrictor response to sympathetic activation to be unchanged following an acute (∼30 min) exposure to intermittent hypoxia; however, these findings were limited to males only (Stuckless et al., 2020). Similarly, four weeks of intermittent hypoxia exposure using a hypobaric chamber (3 h per day, 5 days per week) did not alter resting blood pressure or total peripheral resistance in a mixed sex cohort of young athletes (Fu et al., 2007). To expand results to a female cohort, we measured forearm blood flow responses to acute sympathetic activation utilizing the cold pressor test prior to and following an acute (30 min) intermittent hypoxia exposure (of note, prior work has shown the sympathetic response to cold pressor test is unaffected by OSA; (Narkiewicz et al., 1999)).

First, we found, in agreement with Stuckless and colleagues, that forearm vasoconstriction in response to sympathetic activation is unchanged in healthy young males following 30 min of intermittent hypoxia (Jacob et al., 2023). Second, we presented novel findings in females, whereby forearm vasoconstriction in response to the cold pressor test was blunted following intermittent hypoxia, indicative of a sympatholytic response. In contrast to pre‐clinical data, these observations in females were independent of endogenous sex hormones as participants were studied during both the low (early follicular) and the high (late follicular) hormone phase of the menstrual cycle and elicited similar responses (Jacob et al., 2023). These data suggest the ‘protective effect’ of female sex on sympathetic‐mediated vasoconstriction following intermittent hypoxia exposure persists when endogenous hormones are low (i.e., early follicular phase). Cumulatively, results support sex‐related differences in the vascular response to sympathetic activation following various acute intermittent hypoxia exposures, with more work needed in elucidating the role of sex hormones in humans.

## 3 STEADY‐STATE HYPOXIA

Whereas intermittent hypoxia (e.g., OSA) elicits cyclical blood oxygen desaturations and re‐saturations that promote sympathoexcitation and peripheral vasoconstriction, steady‐state hypoxia elicits a prolonged blood oxygen desaturation that results in a paradoxical peripheral vasodilation to preserve blood flow (Casey et al., 2014). Vascular tone of the skeletal muscle arterioles, essential to blood pressure control, is dictated by the net balance of vasoconstrictor activity and local metabolic vasodilatory influences. Despite an increase in MSNA during steady‐state hypoxia, local skeletal muscle vasodilation predominates (Joyner & Casey, 2014), suggesting factors governing local metabolic vasodilation outcompete neural vasoconstrictor drive during steady‐state hypoxia.

It was historically believed that the vasoconstrictor response to sympathetic activation was blunted during hypoxia. In support of this notion, graded hypoxia exposure attenuated the vasoconstrictor responses to exogenous noradrenaline applied to isolated arteries from rabbits (Marriott & Marshall, 1990). Similarly, brachial artery infusion of exogenous noradrenaline elicited less vasoconstriction during hypoxia relative to normoxia in an all‐male human cohort (Heistad & Wheeler, 1970). In contrast, data from a mixed‐sex cohort utilizing intra‐arterial tyramine to evoke endogenous catecholamine release revealed α‐adrenergic receptor responsiveness to be preserved during hypoxia (Dinenno et al., 2003). However, these prior studies failed to account for a critical physiological component – biological sex.

Expanding results to females, our group applied the cold pressor test during steady‐state hypoxaemia to elicit sympathetic‐mediated vasoconstriction (Jacob et al., 2021). Males exhibited a reduction in forearm blood flow in response to the cold pressor test that did not differ between normoxic and hypoxic conditions. In contrast, we observed an attenuation of sympathetic vasoconstriction in healthy young females during hypoxia relative to responses during normoxia (i.e., hypoxic sympatholysis) (Jacob et al., 2021). This result was later replicated in a second cohort of healthy participants (Jacob et al., 2025), and together led us to conclude the presence of sex‐related differences in the vascular responses to sympathetic activity during hypoxia. Indeed, females appear to exhibit a level of vascular protection to sympathetic insults that is not observed in males. Notably, this ‘protective effect’ of the female sex appears lost with increased CVD risk, such as obesity (Boyes et al., 2025) and possibly ageing (Casey et al., 2014). Indeed, data from our group support an attenuation of hypoxic sympatholysis in females with overweight/obesity, as well as a negative association with measures of adiposity (e.g., percentage body fat, android fat, gynoid fat, waist circumference) and hypoxic sympatholysis (Boyes et al., 2025).

## 4 CONTRIBUTING MECHANISMS

Mechanisms contributing to skeletal muscle vasodilation during hypoxia are multifactorial. In vivo studies in humans demonstrate that nitric oxide (NO) plays a substantial role in hypoxic vasodilation (Blitzer et al., 1996). Further, NO‐mediated vasodilation during hypoxia has been shown to be primarily mediated through β‐adrenergic receptors (Dawes et al., 1997). β‐Adrenergic receptors are located on the vascular endothelium and smooth muscle, eliciting smooth muscle relaxation and subsequent vasodilation (Al‐Gburi et al., 2017). Prior work supports up to 50% of hypoxic vasodilation can be attributed to β‐adrenergic receptors (Blauw et al., 1995; Richardson et al., 1967; Weisbrod et al., 2001; Wilkins et al., 2008). During hypoxia, combined β‐adrenergic and NO blockade did not attenuate hypoxic vasodilation more than what was observed with β‐adrenergic receptor inhibition alone (Weisbrod et al., 2001), suggesting β‐adrenergic receptors are upstream of and mediate NO production in response to hypoxia. However, half of the studies examining β‐adrenergic and hypoxic dilation did not include female participants (Blauw et al., 1995; Richardson et al., 1967) and the other half are from predominantly male cohorts (Weisbrod et al., 2001; Wilkins et al., 2008). Notably, data from male OSA patients support functional downregulation of vascular α‐ and β‐adrenergic receptors (Grote et al., 2000). Considering healthy young females have greater in vivo β‐adrenergic receptor sensitivity relative to males (Kneale et al., 2000) and greater β_1_‐ and β_3_‐adrenergic receptor expression has been observed in isolated arteries from females (Riedel et al., 2019), β‐adrenergic receptors represent a plausible mechanism whereby sex‐related differences occur in the vascular response to hypoxia.

Recent data from our group tested this hypothesis and observed a robust reduction in hypoxic vasodilation when β‐adrenergic receptors were blocked with oral propranolol; notably, this reduction was significant in females only (Jacob et al., 2025). These data demonstrate females, but not males, are reliant on β‐adrenergic receptors to achieve vasodilation in response to hypoxia. However, no effect of β‐adrenergic blockade on sympathetic‐mediated vasoconstriction during hypoxia was observed in either sex (Jacob et al., 2025). Consistent with this, work in exercising rodents observed greater attenuation of sympathetic vasoconstriction during exercise (i.e., functional sympatholysis) in females relative to males; however, β‐adrenergic receptors were not obligatory for this effect (Cooper et al., 2021). In another study by the same group, when NO synthase was inhibited, sympathetic‐mediated vasoconstriction was partly restored in female rodents (Just & DeLorey, 2017). In humans, NO has indeed been shown to directly oppose sympathetic‐mediated vasoconstriction (Shabeeh et al., 2013), and young females exhibit greater vascular and whole body production of NO relative to males (Forte et al., 1998; Kneale et al., 1997). Additionally, in hypertensive rodents, increasing baseline NO reduces sympathetic hyperactivity (Guimarães et al., 2019). Given that oestrogen status is positively correlated with endothelial NO synthase (Gavin et al., 2009), NO represents a potential unexplored mechanism in mediating sex‐related differences in the vascular response to hypoxia. In support of this, exogenous sex hormones appear to influence vascular control in young females; β‐adrenergic receptor‐mediated vasodilation is greater in females using oral hormonal contraceptives (i.e., exogenous ovarian hormones) (Limberg et al., 2016). Further, females taking oral contraceptives exhibit paradoxical peripheral vasodilation during sympathetic activation via a cold pressor test (Jacob et al., 2022) and attenuated vasoconstriction in response to bursts of muscle sympathetic nerve activity (i.e., reduced sympathetic vascular transduction) (D'Souza et al., 2025). Thus, future work should investigate the mechanistic roles of exogenous hormones and β‐adrenergic receptors on the vascular response to hypoxia in females.

## 5 HYPOXIA, HORMESIS AND SEX‐DIFFERENCES

The term ‘hormesis’ is used to describe a bi‐phasic, dose‐dependent response whereby low doses incite beneficial adaptive responses, yet high doses may be maladaptive or harmful. Such is the case with intermittent hypoxia (Burtscher et al., 2024; Puri et al., 2021). Despite an association between OSA and CVD, when intermittent hypoxia is administered acutely, at a moderate dose and in a controlled environment, it may prevent inactivity‐induced endothelial dysfunction (Hanson et al., 2022), enhance shear‐mediated dilation of the carotid artery (Iwamoto et al., 2020), and lower blood pressure in adults with hypertension (Lyamina et al., 2011). Further, in both young and older adults, acute intermittent hypoxia may attenuate ischaemia–reperfusion injury (Jarrard et al., 2021; Stray‐Gundersen et al., 2022).

Based on observed sex‐related differences in the acute neurovascular response to intermittent hypoxia, as outlined above, it is reasonable to propose the hormetic effects of intermittent hypoxia may also differ by sex and/or sex hormone state. To begin to address this possibility, we examined flow mediated vasodilation (FMD, a measure of vascular endothelial function) in males and females following acute (30 min), mild intermittent hypoxia. First, we observed an increase in blood pressure in males, but not females (Mascone et al., 2025) – in alignment with our previous work (Jacob et al., 2021). Despite an increase in blood pressure, there was no change in FMD in male participants after acute intermittent hypoxia (Mascone et al., 2025). Although initially surprising, data from a majority‐male sample similarly showed no change in FMD following intermittent hypoxia (Hanson et al., 2022). Inclusion of a time‐matched control (where participants remained sedentary) uncovered reductions in FMD in the time‐matched sedentary condition which were prevented by intermittent hypoxia (Hanson et al., 2022). Indeed, vascular shear rate increased during intermittent hypoxia, which likely underpinned the preservation of FMD after intermittent hypoxia but not the time‐matched sedentary control (Hanson et al., 2022). Taken together, elevated vascular shear rate during intermittent hypoxia has the potential to offset decreases in FMD experienced during sedentary behaviour, at least in males. In the absence of changes in blood pressure following intermittent hypoxia in females, we observed declines in FMD following intermittent hypoxia exposure (Mascone et al., 2025). We speculate a decline in FMD in females following intermittent hypoxia may be attributable to a steady decline in arterial shear rate (Mascone et al., 2025).

Together our data support sex‐specific effects of intermittent hypoxia on vascular function, whereby acute, mild doses of intermittent hypoxia are associated with potentially beneficial hormetic vascular effects in young males but not young females, likely through blood pressure and shear rate‐related mechanisms. It is unclear how obesity, ageing or sex hormone fluctuations may alter the hormetic effects of intermittent hypoxia on vascular function. Future investigations of these relationships are warranted to uncover any potential clinical benefits of intermittent hypoxia in conditions associated with vascular dysfunction.

## 6 CONCLUSION

**FIGURE 1:**
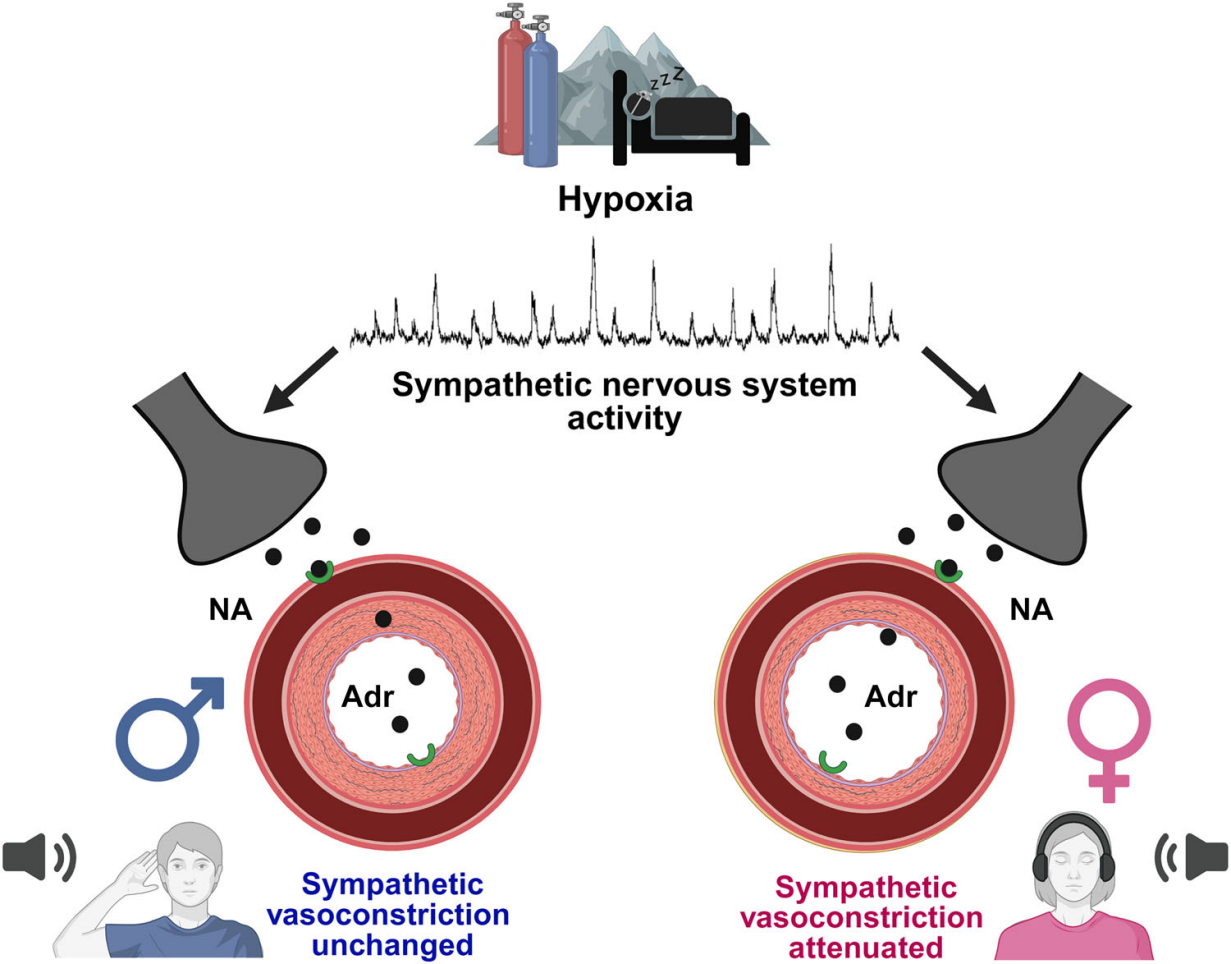
Conceptual diagram. Herein we outline literature supporting sex‐related differences in the acute neurovascular response to intermittent and steady‐state hypoxia. Hypoxia elicits an increase in sympathetic nervous system activity, which promotes neurotransmitter release (NA, noradrenaline, Adr, adrenaline). These neurotransmitters bind to adrenergic receptors, which ultimately results in vasoconstriction. Although the increase in muscle sympathetic nerve activity (MSNA) following intermittent hypoxia exposure, as well as the peak MSNA response to hypoxia, does not differ by sex, the vascular response to sympathetic activation following hypoxic exposure is attenuated in healthy young females, reflecting a potentially beneficial response not observed in healthy young males, which may be lost with obesity and/or ageing.

Herein we outline literature in support of vascular sex‐differences in the acute response to both intermittent and steady‐state hypoxia, with an emphasis on the healthy state (Figure 1). However, questions remain to bridge the gap between preclinical observations and pathological conditions, such as the role of sex hormones and adrenergic receptors, as well as increasing adiposity, age and chronic disease. The present summary of recent literature with emphasis on work from our group(s) may spark new research ideas to champion the cause of alleviating disproportionately poor outcomes in females in conditions where hypoxia is observed.

## AUTHOR CONTRIBUTIONS

Dain W. Jacob, Sara E. Mascone, Natasha G. Boyes, and Jacqueline K. Limberg contributed to the conception and design of the work. Dain W. Jacob, Sara E. Mascone, Natasha G. Boyes, Anna M. Gonsalves, and Jacqueline K. Limberg contributed to acquisition, analysis, and interpretation of data. Dain W. Jacob, Sara E. Mascone, Natasha G. Boyes, Anna M. Gonsalves, and Jacqueline K. Limberg contributed to drafting and/or revising critically for important intellectual content. All authors approved of the final version of the manuscript and agree to be accountable for all aspects of the work in ensuring that questions related to the accuracy or integrity of any part of the work are appropriately investigated and resolved. All persons designated as authors qualify for authorship, and all those who qualify for authorship are listed.

## CONFLICT OF INTEREST

DAIN W. JACOB is a paid research consultant for AbbVie. The other authors declare no conflicts of interest.
